# Ductal adenocarcinoma of the prostate: A systematic review and meta‐analysis of incidence, presentation, prognosis, and management

**DOI:** 10.1002/bco2.60

**Published:** 2021-01-05

**Authors:** Nithesh Ranasinha, Altan Omer, Yiannis Philippou, Eli Harriss, Lucy Davies, Ken Chow, Paolo M. Chetta, Andrew Erickson, Timothy Rajakumar, Ian G. Mills, Richard J. Bryant, Freddie C. Hamdy, Declan G. Murphy, Massimo Loda, Christopher M. Hovens, Niall M. Corcoran, Clare Verrill, Alastair D. Lamb

**Affiliations:** ^1^ Nuffield Department of Surgical Sciences University of Oxford Oxford UK; ^2^ Department of Urology Oxford University Hospitals NHS Foundation Trust, Roosevelt Drive Oxford UK; ^3^ Bodleian Health Care Libraries University of Oxford Oxford UK; ^4^ Department of Surgery Royal Melbourne Hospital University of Melbourne Melbourne VIC Australia; ^5^ Dana Farber Cancer Institute Harvard MA USA; ^6^ Division of Cancer Surgery Peter MacCallum Cancer Centre Melbourne VIC Australia; ^7^ Sir Peter MacCallum Department of Oncology University of Melbourne Parkville VIC Australia; ^8^ Weill Cornell Medical School New York NY USA; ^9^ NIHR Oxford Biomedical Research Centre University of Oxford, John Radcliffe Hospital Oxford UK

**Keywords:** acinar carcinoma, ductal carcinoma, prostate cancer, recurrence, survival

## Abstract

**Context:**

Ductal adenocarcinoma (DAC) is relatively rare, but is nonetheless the second most common subtype of prostate cancer. First described in 1967, opinion is still divided regarding its biology, prognosis, and outcome.

**Objectives:**

To systematically interrogate the literature to clarify the epidemiology, diagnosis, management, progression, and survival statistics of DAC.

**Materials and methods:**

We conducted a literature search of five medical databases from inception to May 04 2020 according to PRISMA criteria using search terms “prostate ductal adenocarcinoma” OR “endometriod adenocarcinoma of prostate” and variations of each.

**Results:**

Some 114 studies were eligible for inclusion, presenting 2 907 170 prostate cancer cases, of which 5911 were DAC. [Correction added on 16 January 2021 after the first online publication: the preceding statement has been corrected in this current version.] DAC accounts for 0.17% of prostate cancer on meta‐analysis (range 0.0837%‐13.4%). The majority of DAC cases were admixed with predominant acinar adenocarcinoma (AAC). Median Prostate Specific Antigen at diagnosis ranged from 4.2 to 9.6 ng/mL in the case series.

DAC was more likely to present as T3 (RR1.71; 95%CI 1.53‐1.91) and T4 (RR7.56; 95%CI 5.19‐11.01) stages, with far higher likelihood of metastatic disease (RR4.62; 95%CI 3.84‐5.56; all *P*‐values < .0001), compared to AAC. Common first treatments included surgery (radical prostatectomy (RP) or cystoprostatectomy for select cases) or radiotherapy (RT) for localized disease, and hormonal or chemo‐therapy for metastatic disease. Few studies compared RP and RT modalities, and those that did present mixed findings, although cancer‐specific survival rates seem worse after RP.

Biochemical recurrence rates were increased with DAC compared to AAC. Additionally, DAC metastasized to unusual sites, including penile and peritoneal metastases. Where compared, all studies reported worse survival for DAC compared to AAC.

**Conclusion:**

When drawing conclusions about DAC it is important to note the heterogenous nature of the data. DAC is often diagnosed incidentally post‐treatment, perhaps due to lack of a single, universally applied histopathological definition. As such, DAC is likely underreported in clinical practice and the literature. Poorer prognosis and outcomes for DAC compared to AAC merit further research into genetic composition, evolution, diagnosis, and treatment of this surprisingly common prostate cancer sub‐type.

**Patient summary:**

Ductal prostate cancer is a rare but important form of prostate cancer. This review demonstrates that it tends to be more serious at detection and more likely to spread to unusual parts of the body. Overall survival is worse with this type of prostate cancer and urologists need to be aware of the presence of ductal prostate cancer to alter management decisions and follow‐up.

**Abbreviations**
DAC: Ductal Adenocarcinoma
AAC: Acinar Adenocarcinoma
PSA: Prostate Specific Antigen
RP: Radical Prostatectomy
RT: Radiotherapy
IDC‐P: Intra‐ductal Adenocarcinoma of the Prostate
PIN: Prostatic Intraepithelial Neoplasia
ISUP: International Society of Urological Pathology
CSS: Cancer‐specific Survival
OS: Overall Survival
BCR: Biochemical Survival
SEER: Surveillance, Epidemiology and End Results Programme
TURP: Trans‐Urethral Resection of the Prostate
TRUS: Trans‐Rectal Ultra Sound
MRI: Magnetic Resonance Imaging
PI‐RADS: Prosatate Imaging Reporting and Data System
IHC: Immunohistochemistry
ADT: Androgen Deprivation Therapy
PSMA: Prostate Specific Membrane Antigen
PET: Positron Emission Tomography
RARP: Robot‐Assissted Radical Prostatectomy

## 1 INTRODUCTION

Acinar adenocarcinoma (AAC) is the main subtype of prostate carcinoma, accounting for over 90% of all primary carcinomas of the prostate.^1^ First described in 1967 by Melicow and Pachter,^2^ ductal adenocarcinoma (DAC) is relatively rare, yet is nonetheless the second most common subtype of prostatic carcinoma. Previously also known as “endometrioid” or “papillary” carcinoma, DAC was initially believed to be of endometrial origin, arising in the verumontanum.

Although more than 50 years have elapsed since DAC was first reported, opinion is still divided regarding its biology, diagnosis, and outcome. Recent reviews on DAC focus predominantly on genetic and histological differences between DAC and other subtypes, with a view to improving diagnostic success and better understanding disease progression.^3^, ^4^, ^5^, ^6^ However there is little consensus regarding the common management strategies, disease progression, and outcomes. In this review, we systematically interrogated the literature, present updated epidemiological data, and focus particularly on the distinct management strategies that must be employed with DAC as compared with regular AAC, and assess specifically the behavior of DAC in terms of clinical outcomes.

## 2 MATERIALS AND METHODS

### 2.1 Search strategy

The following databases were searched from inception to 4th May 2020 for relevant studies: PubMed; Ovid EMBASE; the Cochrane Library; SCOPUS; and the Web of Science Core Collection. The literature search combined the thesaurus terms Carcinoma, Ductal/ AND Prostatic Neoplasms/ where relevant, along with a set of phrases combined with OR to retrieve references, for example, about “ductal adenocarcinoma of the prostate,” “ductal carcinoma of the prostate,” or “ductal prostate cancer.” We did not use any limits. The full search strategies are available in the appendix (Supporting Document S1) and have been registered with PROSPERO (CRD42019122205) 16 August 2019 (Supporting Document S2).

### 2.2 Inclusion and exclusion criteria

**FIGURE 1:**
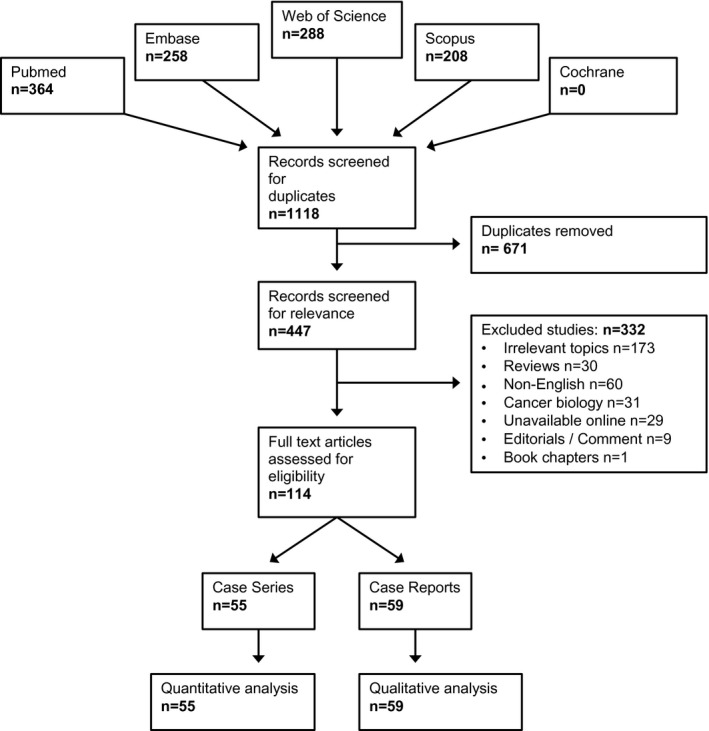
PRISMA flowchart

De‐duplicated studies were screened for relevance, of which 114 were eligible for inclusion (Document S3). These studies were divided into case reports (<3 cases) and case series (3 + cases). The excluded studies met at least one of the following criteria: (i) the article was a review or meta‐analysis, editorial comment, letter or book chapter (ii) non‐English language (iii) a basic science cancer biology article (iv) unavailable (Figure 1). Intra‐ductal adenocarcinoma of the prostate (IDC‐P) and prostatic intraepithelial neoplasia (PIN)‐like ductal carcinoma of the prostate were both excluded as separate histopathological entities to DAC.

### 2.3 Data extraction

The data were extracted from full‐length articles (and abstracts where full‐length articles were unavailable) by two reviewers, NR, and AO (Table S1). The data included the author's name, city and country, the year and journal of publication, number of DAC cases reported (with comparative AAC cases where available) and number of pure versus mixed cases, time period covered, a pathological definition of the tumor, the patients’ age, International Society of Urological Pathology (ISUP) score and Prostate Specific Antigen (PSA), method of DAC diagnosis, staging including positive lymph nodes and distant metastases on initial presentation, primary treatment modality, and post‐treatment outcomes (including post‐treatment metastases and results of follow‐up treatments).

### 2.4 Data analysis and synthesis

The case reports were included in qualitative analysis while quantitative analysis was limited to the case series. There was heterogeneity in terms of disease description and outcome reporting among the case series and hence meta‐analysis was limited to only those studies relevant for each outcome analyzed (eg: PSA; Cancer‐specific Survival; Overall Survival; Biochemical Survival [BCR]). Where relevant, relative risk was calculated using Revman statistical software. Primary outcomes investigated included incidence, PSA at diagnosis, T,N,M stage at diagnosis, BCR after RT and RP treatment, and cancer‐specific and overall survival (Table S2).

### 2.5 Risk of bias analysis

All studies included in the analysis were screened with the ROBINS‐E risk of bias (ROB) tool (Table S3).

## 3 RESULTS

A total of 114 publications were eligible for inclusion, comprising 2 907 170 cases of prostate cancer, of which 5911 were DAC. [Correction added on 16 January 2021 after the first online publication: the preceding statement has been corrected in this current version.] This consisted of 55 case series and 59 case reports (Figure 1, Table S1). All of these studies were retrospective with only a few comparing the outcomes for DAC to AAC.

### 3.1 Epidemiology

Of the 55 case series, 21 studies presented consecutive counts of both DAC and AAC enabling calculation of DAC incidence, which was 0.17% on meta‐analysis (range 0.0837%‐13.4%) (Table 1). The small number case reports or selective case series were excluded. A handful of studies retrospectively analyzed the National Cancer Database and Surveillance, Epidemiology and End Results Programme (SEER) cancer registry to extract large samples.^7^, ^8^, ^9^ For example, Packiam 2015 et al identified 1328 DAC cases out of 716 963 AAC cases at an incidence of 0.19%.^10^ Our findings are consistent with this large retrospective study.

**TABLE 1:** Presenting incidence of ductal adenocarcinoma (DAC) from consecutive case series.

Study	DAC	AAC	Total
Iakymenko, 2019^42^	128	1141	1269
Knipper, 2019^52^	581	489 296	489 877
Jang, 2017^50^	101	2547	2648
Wu, 2017^40^	511	3303	3814
Zargar, 2016^53^	12	2276	2288
Mathur, 2016^54^	54	9892	9946
Packiam, 2015^10^	1328	715 635	716 963
Kim, 2015^55^	33	3947	3980
Gulavita, 2015^21^	46	1081	1127
Seipel, 2014^23^	69	982	1051
Tarjan, 2012^29^	13	97	110
Meeks, 2012^9^	693	737 262	737 955
Amin, 2011^48^	93	18 459	18 552
Samaratunga, 2010^49^	34	234	268
Morgan, 2010^7^	371	442 881	443 252
Kendal, 2010^8^	642	450 743	451 385
Tu, 2009^47^	108	13 017	13 125
Eade, 2007^56^	6	4515	4521
Bock, 1999^57^	17	321	338
Lee, 1994^58^	6	1576	1582
Christensen, 1991^59^	15	735	750
**Total**	**4861**	**2 899 940**	**2 904 801**
**Incidence**	**0.17%**		

It has been clear for some time that the majority of cases present admixed, with DAC as the minor component and predominant AAC. In 1975, Tannenbaum reviewed pathology reports at Columbia‐Presbyterian Medical Center and reported “the majority of DAC tumors were associated with conventional acinar carcinomas”.^11^ A more recent retrospective analysis of 1051 radical prostatectomy specimens from Karolinska University Hospital, Sweden in 2013 supported this view quantitatively, demonstrating pure DAC in two specimens (0.2%) and mixed acinar‐ductal adenocarcinoma in 84 specimens (8%).^12^ Sporadic reports describe DAC also coexisting with non‐acinar carcinomas, including urothelial and sarcomatoid carcinoma.^13^, ^14^, ^15^, ^16^ Lee et al identified several rare histological variants of DAC, including foamy gland, mucinous goblet cell and paneth cell‐like DAC.^17^ Clearly, precise definitions in DAC are important.

### 3.2 Definition and diagnosis

#### 3.2.1 PSA value

Mean age at diagnosis ranged from 58 to 78 years. Some 19 case series reported PSA at diagnosis, of which 11 presented median PSA, ranging from 4.2 to 9.6 ng/mL, and 8 presented mean PSA, which was 10.1 ng/mL on meta‐analysis (Table 2). PSA at diagnosis ranged from 0.27 to 130 ng/mL in the case reports. This analysis reveals lower and more variable PSA values for DAC compared to AAC, suggesting PSA may have a more limited role in early detection of DAC. In their large series of 371 DAC cases, Morgan et al support this hypothesis, reporting that ductal histology is associated with a 30% lowering of geometric mean PSA compared to AAC, and a more than two‐fold increased chance of having a PSA < 4 ng/mL, independent of other clinicopathological variables.^7^ In lieu of PSA, Sathiamoorthy proposes using cytomorphologic features in urine cytology to diagnose DAC, since DAC usually presents at an advanced stage and may exfoliate owing to its location in the prostatic central urethra.^18^ Recently, Lin et al supported this proposal in a small study, demonstrating urine cytology was the first evidence of disease in 4/5 (80%) DAC cases compared to 6/23 (26%) AAC cases.^19^

**TABLE 2:** Presenting PSA in DAC

Study	PSA (mcg/L)		No. of cases
	Median	Mean	
Zhi et al, 2017^60^	6.7		31
Zargar et al, 2016^53^	7.8		12
Mathur et al, 2016^54^	9.5		54
Bergamin et al, 2016^61^	9.6		27
Packiam et al, 2015^10^		10.3	1328
Kim et al, 2015^55^		14.7	33
Gulavita et al, 2015^21^		12.9	46
Coffey et al, 2015^62^	5.2		8
Schieda et al, 2014^63^		6	11
Seipel et al, 2013^12^		8.2	86
Tarjan et al, 2012^29^	9		13
Meeks et al, 2012^9^	6.2		693
Amin et al, 2011^48^		7.7	93
Aydin et al, 2010^64^		12.5	23
Samaratunga et al, 2010^49^	8.4		34
Orihuela et al, 2008^20^	5.6		17
Tavora et al, 2008^65^		5.9	28
Eade et al, 2007^56^	4.2		6
Brinker et al, 1999^66^	7.9		58
**Meta‐analysis**	**Min = 4.2**	**Weighted mean = 10.1**	**2601**
	**Max = 9.6**		

Orihuela et al demonstrated that DAC produces less PSA than acinar adenocarcinoma.^20^ In addition to diagnostic implications, this observation also suggests DAC may progress post‐prostatectomy without biochemical recurrence; potentially leading to progression to symptomatic distant metastatic disease and missing the chance for early initiation of salvage treatments.

#### 3.2.2 Histopathology

**FIGURE 2:**
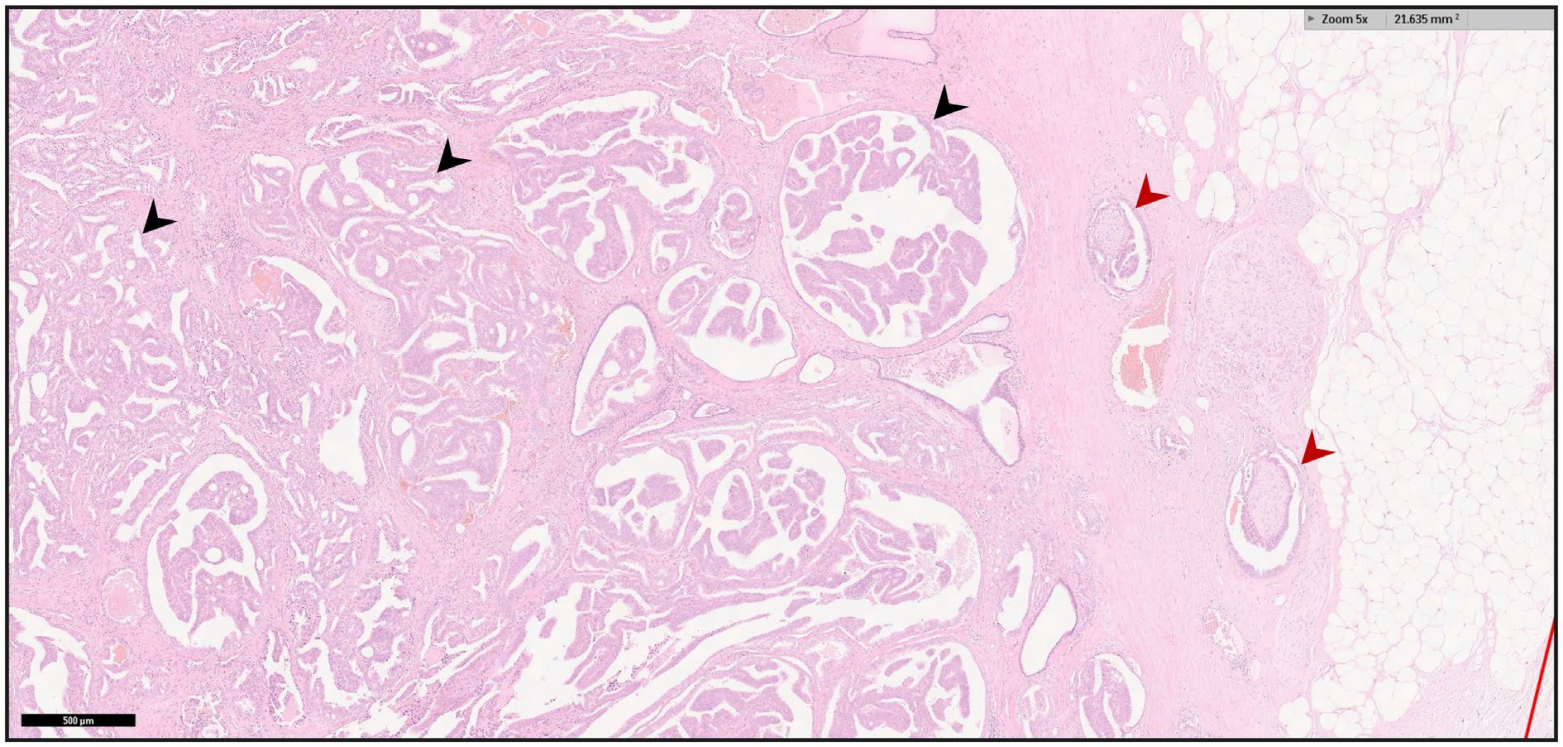
Histology of DAC. Black arrows indicate ductal cancer glands. Red arrow indicates extra‐prostatic extension. Scale bar 500 µm

Given the apparent deleterious prognostic implications of DAC, accurate identification of DAC amongst coexisting subtypes in pre‐treatment reports is important. However, DAC is often diagnosed incidentally on post‐treatment specimen histopathological report (prostatectomy specimen, Trans‐Urethral Resection of the Prostate chippings) rather than on the Trans‐Rectal Ultra Sound (TRUS) biopsy cores. Gulavita et al. highlight this problem, demonstrating only 2/18 cases were correctly diagnosed as DAC on TRUS biopsy, with the remainder only diagnosed from post prostatectomy specimens.^21^ More recently, Prendeville et al used multi‐parametric Magnetic Resonance Imaging (MRI) targeted biopsy to diagnose 23 DAC cases out of 103 prostate carcinomas, commenting that in 83% (19 out of 23 cases) prior 12‐core standard systematic biopsy missed the DAC component entirely.^22^ They further demonstrated an association between the DAC cases and Prosatate Imaging Reporting and Data Sysytem (PI‐RADS) 5 MRI classification, reinforcing the view that DAC is highly likely to be a clinically significant cancer. Typically, DAC is characterized by distinctive tall columnar, pseudostratified epithelium with a papillary, cribriform, glandular, or solid architecture (Figure 2), while AAC comprises cuboidal cells arranged in acini. However, lack of a single, universally applied histopathological definition for DAC is an impediment. Seipel et al demonstrated the inter‐observer variability in DAC diagnosis among 20 expert uropathologists with only 52% consensus for positive identification of DAC.^23^ They also ventured a framework of minimum diagnostic criteria for DAC, identifying papillary architecture with true fibrovascular stalks as the most useful standalone diagnostic feature of DAC. Recently, Au et al studied 45 cases of DAC and demonstrated that DAC with cribriform architecture has a higher likelihood of extra‐prostatic extension, seminal vesicle invasion, lympho‐vascular invasion and advanced pathologic stage than non‐cribriform DAC.^24^ This suggests that DAC with cribriform architecture could be a more aggressive subtype of DAC. However, given current limitations in DAC diagnosis in general, subtyping DAC may be challenging at present and of limited value practically.

Intra‐ductal adenocarcinoma of the prostate (IDC‐P) is perhaps the hardest differential subtype to distinguish from DAC. Like DAC, IDC‐P carries a worse prognosis compared to AAC.^25^, ^26^ In principle, DAC represents a proliferation of in situ ductal cells while IDC‐P represents a proliferation of invasive acinar cells into the duct system. This explains the absolute requirement for a basal cell layer in IDC‐P diagnosis, unlike in DAC. Furthermore, DAC cells are generally smaller and columnar with elongated nuclei while IDC‐P cells are larger and cuboidal with round nuclei.^27^ In practice however, histological differentiation between prostate carcinoma subtypes can be difficult due to overlapping characteristics. Nearly one third of DAC cases show presence of basal cells and intra‐ductal growth by Immunohistochemistry staining.^28^ Seipel et al suggest the presence of nuclear elongation can be helpful in excluding IDC‐P specifically.^23^

#### 3.2.3 T staging

As suggested by multiple retrospective studies, DAC is more likely to present as T3 stage compared to AAC. On meta‐analysis, the percentage of T3 disease is 22.2% in DAC and 8.9% in AAC (Table 3, Figures S4‐S7). As expected, the majority of DAC cases were < T3. DAC is more locally advanced than AAC with a 1.71 (1.53‐1.91) RR for T3 disease and a 7.56 (5.19‐11.01) RR for T4 disease on meta‐analysis. A SEER analysis in 2012 by Meeks et al^9^ supported our analysis, demonstrating DAC was more likely to have an initial clinical diagnosis of T3 disease than AAC, with an incidence of 30.3% versus 18.0%, respectively. Their analysis has led to the conclusion that DAC resembles ISUP Grade Group 3 + AAC, in terms of initial T stage and prognosis. Furthermore, this was consistent with the pathological T stage in another study by Tarjan et al^29^ who found a nearly three‐fold higher incidence for pT3 in DAC versus AAC.

**TABLE 3:** T stage distribution for DAC and AAC

Study	Tumour type	Total cases	T1 (%)	T2 (%)	T3 (%)	T4 (%)
Wu et al, 2017^40^	DAC	511	103 (20.2)	213 (41.0)	115 (22.5)	54 (10.6)
	AAC	3303	446 (13.5)	2256 (68.3)	522 (15.8)	47 (1.4)
Kim et al, 2015^55^	DAC	29	16 (55.2)	6 (20.7)	7 (24.1)	
	AAC	116	68 (56.6)	24 (19.9)	28 (23.3)	
Tarjan et al, 2012^29^	DAC	13	1 (8.0)	1 (8.0)	10 (76.0)	1 (8.0)
	AAC	97	49 (51.0)	22 (23.0)	26 (27.0)	0 (0.0)
Meeks et al, 2012^9^	DAC	293	43 (14.7)	110 (38.0)	89 (30.3)	
	AAC	257 875	77 390 (30.0)	123 919 (48.0)	47 024 (18.0)	
Morgan et al, 2010^7^	DAC	324	130 (40.0)	155 (48.0)	39 (12.0)	
	AAC	414 587	187 893 (45.0)	214 110 (52.0)	12 584 (3.0)	
**Total**	**DAC**	**1170**	**293(25.0)**	**485 (41.5)**	**260 (22.2)**	**55 (10.5)**
	**AAC**	**675 978**	**265 846 (39.3)**	**340 331 (50.4)**	**60 184 (8.9)**	**47 (1.4)**
		**RR**	0.88 (0.80‐0.97)	0.74 (0.69‐0.79)	1.71 (1.53‐1.91)	7.56 (5.19‐11.01)
		***P* value**	*P* = .01	*P* < .00001	*P* < .00001	*P* < .00001

#### 3.2.4 N and M staging

**FIGURE 3:**
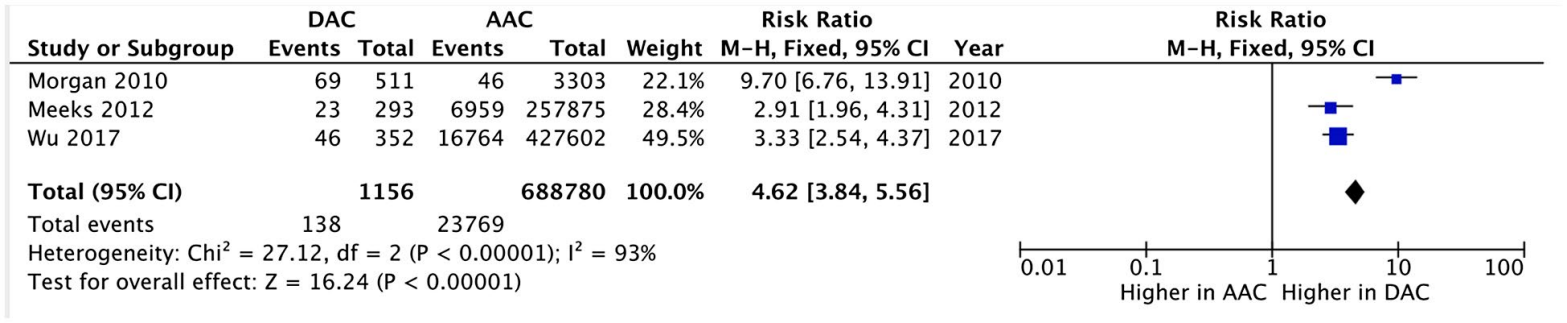
Risk ratio of positive M status at presentation

DAC has a different behavior compared to AAC in terms of both incidence and site of metastasis. On meta‐analysis the relative risk of node and metastasis positive disease at presentation is 1.04 (0.97‐1.11) (Figure S8) and 4.62 (3.84‐5.56) (Table 4, Figure 3) respectively, compared to AAC. Lower PSA at presentation, leading to delayed diagnosis, could contribute to the higher risk of metastases. Supporting our analysis, Meeks et al reviewed 693 DAC cases from 1970 to 2007 in the SEER cancer registry, demonstrating patients with DAC have metastases at a near three‐fold higher incidence than patients with AAC (11% vs 4%; Table 4).^9^

**TABLE 4:** Relative risk of node status and metastasis for DAC and AAC

Study	Tumour type	Total cases	Positive N status (%)	Positive M status (%)
Wu et al, 2017^40^	DAC	511	339 (66.3)	69 (13.5)
	AAC	3303	2149 (65.1)	46 (1.4)
Meeks et al, 2012^9^	DAC	293	6 (3.0)	23 (11.0)
	AAC	257 875	3315 (1.8)	6959 (4.0)
Amin et al, 2011^48^	DAC	93	5 (5.3)	
	AAC	18 459	443 (2.4)	
Morgan et al, 2010^7^	DAC	352		46 (12.0)
	AAC	427 602		16 764 (4.0)
**Total**	**DAC**	**1249**	**350/897 (39.0)**	**138/1156 (11.9)**
	**AAC**	**707 239**	**5907/279637 (2.1)**	**23769/688780 (3.5)**
		**RR**	1.04 (0.97‐1.11)	4.62 (3.84‐5.56)
		***P* value**	*P* = .3	*P* < .00001

While we know that the vast majority of AAC metastasizes to the bone, DAC spreads to visceral organs such as the lungs and brain more often than acinar adenocarcinoma.^30^, ^31^ Several reports of DAC metastases to the penis,^32^ testes,^33^ and skin^34^ also exist. These are particularly noteworthy since metastases to these organs are exceedingly rare in prostate carcinoma. Therefore, logic dictates prostate carcinoma patients with unusual secondary tumors, should undergo evaluation to exclude DAC, even if the morphological appearance of the tumor is suggestive of non‐prostatic origin. Interestingly for those patients with metastasis at presentation DAC does not portend worse survival. Wu et al conducted the first study investigating the prognostic value of DAC in de novo metastatic prostate cancer.^35^ In their cohort of 634 metastatic prostate cancer cases, the 35 DAC cases were not associated with worse overall or cancer‐specific survival.

### 3.3 Treatment

Currently, treatment modalities for DAC are similar to those available for acinar adenocarcinoma. Typically, radical prostatectomy (RP) and radiotherapy (RT) are favored for localized disease, and androgen deprivation therapy (ADT) and chemotherapy are reserved for metastatic disease. In select patients, cystoprostatectomy and urinary diversion may be considered, for example, in patients with extensive T3 disease at the base of prostate, and in those with endoscopic extension of tumor within the prostatic urethra. Selection of the optimal treatment strategy is currently curtailed by an incomplete understanding of DAC biology and behavior, on account of the small numbers of studies so far.

Few studies compared RP and RT treatment modalities (Table 5). One study, Igdem 2010, reported RT to have more favorable biochemical relapse rates and cancer‐specific survival than RP.^36^ Conversely, the same study reported RP to have more favorable overall survival than RT,^36^ although a tendency for selecting RT in unfit men should be considered as a possible confounder here.^37^, ^38^ As such, although there is no clear consensus regarding the optimal treatment modality for DAC, it does seem that radiotherapy may be superior, perhaps because of the reduced likelihood of seeding of ductal cancer cells into the peritoneal cavity as might occur with radical prostatectomy.

**TABLE 5:** Biochemical relapse (BCR), cancer‐specific survival (CSS), and overall survival (OS) for men with DAC according to mode of radical treatment: radical prostatectomy (RP) or radical radiotherapy (RT)

Study	Outcome measure	Endpoint (yrs)	Total cases	RP (%)	RT (%)
Igdem et al, 2010^36^	BCR	5	RP‐16, RT‐14	3 (19.0)	2 (14.0)
	CSS			14 (88.0)	13 (93.0)
	OS			13 (81.0)	9 (64.0)

### 3.4 Prognosis

Since its identification, our understanding of the prognosis of DAC has evolved. Interestingly, DAC was initially thought to have a favorable prognosis because there were no tumor‐related deaths in early reports.^39^ Tannenbaum even went so far as considering DAC a tumor with no metastatic potential.^11^ The hypothesis was that the periurethral location of DAC (compared to the more peripheral location of acinar adenocarcinoma) led to early diagnosis due to clinical symptoms haematuria as well as lower urinary tract obstructive and irritative symptoms. Accordingly, the tumors may have been treated at an earlier stage prior to metastasis, leading to a more favorable outcome.

However, further studies clearly identified DAC as an aggressive, invasive subtype of prostate carcinoma with overtly worse outcomes than AAC^40^ (Table 6, Figures S1‐S3). On meta‐analysis survival rates, both cancer‐specific (RR0.85 (0.82‐0.88)) and overall (RR0.83 (0.81‐0.85)), were less favorable for DAC compared to AAC 5 years post radical treatment. This could be due to the higher tumor grade at outset, or indeed to the central location in the prostate, allowing occult growth and metastasis. Bostwick et al showed 5‐year survival to be only 15%, with > 50% of patients dying of metastatic disease within 9‐70 months of diagnosis.^41^ Wu et al analyzed 511 DAC cases from the SEER cancer registry, demonstrating improvement in 5‐year cancer‐specific survival to 72%, but was nevertheless still 20% less than AAC.^40^ Furthermore, DAC was shown, on average, to be larger, with a higher pathological stage at diagnosis, than AAC. Iakymenko et al recently demonstrated that, when controlled for tumor grade and volume, DAC did not have an independent effect on short‐term radical prostatectomy outcomes, including positive surgical margins, extra‐prostatic extension and seminal vesicle invasion.^42^

**TABLE 6:** Relative risk of biochemical relapse (BCR), cancer‐specific survival (CSS) and overall survival (OS) with DAC vs AAC

Study	Tumour type	No. cases	BCR @5 yrs (%)	CSS @5 yrs (%)	OS @5 yrs (%)
Wu et al, 2017^40^	DAC	511		370 (72.0)	
	AAC	3303		3071 (93.0)	
Packiam et al, 2015^10^	DAC	1328			996 (75.0)
	AAC	751 635			681 919 (77.0)
Tarjan et al, 2012^29^	DAC	13	8 (62.0)	12 (92.0)	12 (92.0)
	AAC	97	11 (11.0)	97 (100.0)	94 (97.0)
Meeks et al, 2012^9^	DAC	435		383 (88.0)	318 (73.0)
	AAC	442 169		424 482 (96.0)	367 000 (83.0)
**Total**	**DAC**	**2287**	**8/13 (62.0)**	**765/959 (79.8)**	**1326/1776 (74.7)**
	**AAC**	**1 197 204**	**11/97 (11.0)**	**427650/445568 (96.0)**	**1049013/1193901 (87.9)**
		**RR (CI)**	**5.43 (2.69‐10.96)**	**0.85 (0.82‐0.88)**	**0.83 (0.81‐0.85)**
		*P* value	*P* < .00001	*P* < .00001	*P* < .00001

#### 3.4.1 Genetics

Profiling the genetic landscape of DAC is a relatively recent endeavor with potential to revolutionize global understanding of DAC. Seipel et al demonstrated the overall level of somatic alterations in DAC is similar to high grade AAC.^43^ Furthermore, they identified DAC harbored somatic changes seen in metastatic castration‐resistant AAC.^43^ Whole exome sequencing of coincident ductal and acinar carcinomas suggest they are derived from a common progenitor, with prognostic divergence possibly driven by varied accumulation of PTEN or CTNNB1 alterations.^44^ Vinceneux et al recently found increased cell proliferation rate and PTEN loss in pure DAC than in high‐grade acinar adenocarcinoma matched for pathological stage.^45^ Ductal pathology is more common in patients with germline DNA‐repair defects, and mismatch repair alterations have been identified in up to 50% of tumors.^46^ These findings help account for the poor prognosis of DAC.

#### 3.4.2 Post‐operative metastatic spread

Tarjan et al demonstrate a higher rate of post‐treatment metastatic spread in DAC, compared to AAC.^29^ Interestingly, in some cases where the primary prostatic carcinoma was identified as mixed acinar‐ductal carcinoma, the secondary metastasis was found to be pure DAC, suggesting a dominant aggressive role for the DAC component as the “index lesion” in the primary. Further investigation into the underlying genetics of DAC and mechanisms of metastasis should improve survival in such men. There may even be a “field‐effect” created by DAC to alter behavior of neighboring AAC, which could explain the findings of Tu et al, who appeared to demonstrate that the prognosis for mixed acinar‐ductal adenocarcinoma was less favorable after radical prostatectomy than pure DAC, with mean survivals of 8.9 and 13.9 years respectively.^47^ In this study, although the median time to local progression was shorter (2.8 vs 4.9 years), the median time to distant metastases was longer (3.9 vs 2.0 years) for patients with pure DAC than mixed acinar‐ductal DAC. The results of this retrospective review of 108 cases suggest that mixed acinar‐ductal adenocarcinoma pursues a more aggressive course than pure DAC. Prospective studies are needed to confirm this unusual finding.

Unusually, nine cases report peritoneal metastases,^20^, ^31^ although the reasons for this are unclear (see Section 4.1).

#### 3.4.3 Ductal proportion

The observation that mixed acinar‐ductal carcinoma has a distinct prognosis independent of its pure variants raises an important question; does the proportion of the ductal component in mixed acinar‐ductal adenocarcinoma affect oncological outcomes? Amin et al showed that mixed acinar‐ductal adenocarcinomas with a ductal component <10% have a prognosis analogous to pure AAC.^48^ Samaratunga demonstrated that any proportion of DAC predicts extra‐prostatic extension.^49^ Jang et al investigated this correlation in a 2016 retrospective review of 101 cases of mixed acinar‐ductal adenocarcinoma between 2005 and 14.^50^ They stratified these cases into a high ductal component group (>30% ductal) and a low ductal component group (<30% ductal) and measured risk of biochemical recurrence in each group. Freedom from biochemical recurrence was significantly lower in the high ductal group than the low ductal group. A high ductal component was also a significant predictor for biochemical recurrence. Recently, Harkin demonstrated this correlation more precisely, showing that, in 68 DAC cases, risk of biochemical recurrence increased linearly per 10% ductal component up to 50% and more substantially beyond 50%.^51^ These results suggest proportion of DAC could potentially be used as a surrogate for poor prognosis or as a determinant for adjuvant therapy. Given the roughly 10‐fold greater incidence and less favorable prognosis of mixed acinar‐ductal adenocarcinoma than pure DAC, the insights from these studies appear particularly relevant. However, it should be noted that Seipel failed to demonstrate any correlation between proportion of DAC and rate of biochemical recurrence in their analysis of 84 mixed acinar‐ductal adenocarcinomas.^12^

## 4 DISCUSSION

### 4.1 Recommendations and limitations

We find the increased likelihood of peritoneal metastases after treatment of ductal adenocarcinoma with radical prostatectomy (Section 3.4.2) to be particularly noteworthy. Is this perhaps because of the opportunity for seeding during radical prostatectomy? Compared to more peripheral AAC, peri‐urethral DAC cells are more likely to extravasate from ducts into the peritoneum during urethral dissection and prostate manipulation in surgery. We hypothesize that radiotherapy or perhaps radical cystoprostatectomy may be more suitable than conventional radical prostatectomy, in avoiding this potentially iatrogenic complication. There is certainly an opportunity for assessment of this intervention and outcome in the setting of a randomized trial, although such a trial would be difficult given the relatively infrequent nature of DAC and the fact that it is often not detected until inspection of the surgical specimen, rather than at diagnostic biopsy.

**FIGURE 4:**
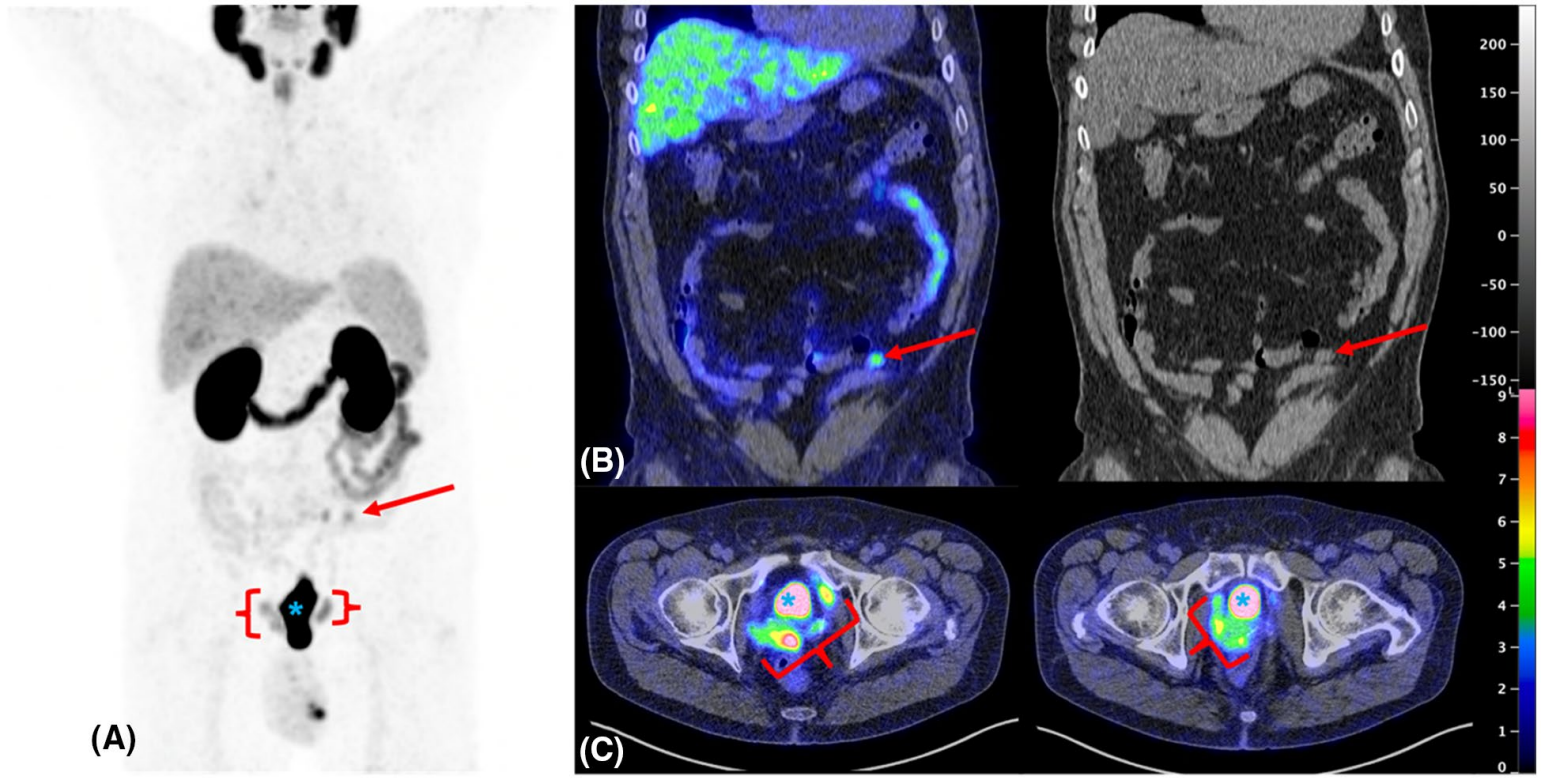
68Gallium PSMA‐PET of pelvic and abdominal disease 6 months after robot‐assisted radical prostatectomy (RARP). A clear mesenteric/peritoneal metastasis is visible (panel A & B, red arrow), alongside bulky pelvic disease (panel C, red bracket)

Additionally, in light of increased post‐operative metastatic spread with DAC, and the possibility of lower PSA production (Section 3.2.1), it may be beneficial to monitor such men with imaging (MP‐MRI ± Prostate Specific Membrane Antigen [PSMA]‐Positron Emission Tomography [PET]) in addition to PSA (Figure 4).

This review, particularly the meta‐analyzed components, have some limitations. In trying to encompass all papers published on this topic we have, inevitably, very heterogeneous source data (Table S3). Perhaps due to the relatively low incidence compared to acinar prostate cancer (0.19% of all prostate cancer) and difficulty in diagnosis, no randomized control trials or prospective cohort studies investigate DAC. The best available evidence is currently limited to case reports and case series, with heterogeneity of disease description and outcome reporting. There is also the possibility of “double counting” where more than one set of authors have accessed the same database.^7^, ^9^, ^40^ As such, we advise caution in interpretation of our meta‐analyses components and also any recommendations we make.

## 5 CONCLUSION

When drawing conclusions about DAC it is important to note the heterogenous nature of the data. DAC is rare but the second most common sub‐type of adenocarcinoma of the prostate, after AAC, occurring in 0.17% of cases. It generally presents with a lower PSA, higher T stage, and higher ISUP Grade Group than AAC. It more commonly gives rise to lymph nodes spread and metastases, with metastases appearing in unusual locations compared to the normal skeletal and nodal spread of AAC, particularly after prostatectomy when it has been noted to undergo disseminated intraperitoneal metastasis, perhaps because of the disruption of prostatic ducts and disconnection of bladder and urethra during surgery. For this reason radiotherapy, to which DAC responds well, or even radical cystoprostatectomy might be better considered in cases of DAC, with radical prostatectomy perhaps unwise due to seeding on opening the bladder neck. Rates of metastasis and cancer‐specific survival are notably worse after DAC compared to AAC, as is BCR, and the generally lower PSA levels in ductal cancer could limit the efficacy of PSA‐based follow‐up.

## Supporting information

Table S1

Table S2

Supplementary Material

Table S3
