# Supplementary material for: Ductal adenocarcinoma of the prostate: A systematic review and meta‐analysis of incidence, presentation, prognosis, and management
Source: BJUI Compass. 2021 Jan 5;2(1):13–23. doi: 10.1002/bco2.60 (PMC8988764; doi:10.1002/bco2.60)
Supplement: Supplementary file 2 — Table S2 [file BCO2-2-13-s002.pdf]

Author(s):
Question: DAC compared to AAC for PCa
Setting:
Bibliography:

Certainty assessment							№ of patients		Effect		Certainty	Importance
**№ of studies**	**Study design**	**Risk of bias**	**Inconsistency**	**Indirectness**	**Imprecision**	**Other considerations**	**DAC**	**AAC**	**Relative (95% CI)**	**Absolute (95% CI)**		
**BCR (follow up: mean 5 years)**												
1	observational studies	serious [a]	not serious	not serious	very serious [b,c]	publication bias strongly suspected very strong association [d]	8/13 (61.5%)	11/97 (11.3%)	**RR 5.42** (2.69 to 10.97)	**501 more per 1,000** (from 192 more to 1,000 more)	⨁◯◯◯ VERY LOW	CRITICAL
**Overall survival (follow up: mean 5 years; assessed with: death)**												
3	observational studies	serious [e]	not serious	not serious	not serious	none	1326/1776 (74.7%)	1049013/1193901 (87.9%)	**RR 0.83** (0.81 to 0.85)	**149 fewer per 1,000** (from 167 fewer to 132 fewer)	⨁◯◯◯ VERY LOW	CRITICAL
**Cancer specific survival (follow up: mean 5 years; assessed with: death)**												
3	observational studies	not serious	not serious	not serious	not serious	none	765/959 (79.8%)	427650/445568 (96.0%)	**RR 0.85** (0.82 to 0.88)	**144 fewer per 1,000** (from 173 fewer to 115 fewer)	⨁⨁◯◯ LOW	CRITICAL
**Positive T3 stage**												
5	observational studies	not serious	not serious	not serious	not serious	strong association	260/1170 (22.2%)	60184/675978 (8.9%)	**RR 1.71** (1.53 to 1.91)	**63 more per 1,000** (from 47 more to 81 more)	⨁⨁⨁◯ MODERATE	CRITICAL
**Positive N status**												
3	observational studies	serious [e]	not serious	not serious	very serious [f]	none	350/897 (39.0%)	5907/279637 (2.1%)	**RR 1.04** (0.97 to 1.11)	**1 more per 1,000** (from 1 fewer to 2 more)	⨁◯◯◯ VERY LOW	IMPORTANT
**Positive M status**												

3	observational studies	not serious	not serious	not serious	not serious	very strong association	138/1156 (11.9%)	23769/688780 (3.5%)	**RR 4.62** (3.84 to 5.56)	**125 more per 1,000** (from 98 more to 157 more)	⨁⨁⨁⨁ HIGH	CRITICAL

CI: Confidence interval; **RR:** Risk ratio

Explanations

a. serious risk of confounder bias
b. small sample size
c. wide confidence interval
d. small study showing large effect
e. serious risk of bias in selection of the reported result in one study
f. RR is 1.04, confidence interval spans 1
