# Supplementary material for: Ductal adenocarcinoma of the prostate: A systematic review and meta‐analysis of incidence, presentation, prognosis, and management
Source: BJUI Compass. 2021 Jan 5;2(1):13–23. doi: 10.1002/bco2.60 (PMC8988764; doi:10.1002/bco2.60)
Supplement: Supplementary file 3 — Supplementary Material [file BCO2-2-13-s004.docx]

**Supplementary Material**

**Ductal adenocarcinoma of the prostate: A systematic review and meta-analysis of incidence, presentation, prognosis and management**

Nithesh Ranasinha^1,2^, Altan Omer^1^, Yiannis Philippou^1^, Eli Harriss^3^, Lucy Davies^1^, Ken Chow^4^, Paolo M Chetta^5^, Andrew Erickson^1^, Timothy Rajakumar^1^, Ian G Mills^1^, Richard Bryant^1,2^, Freddie Hamdy^1,2^, Declan G Murphy^6,7^, Massimo Loda^5,8^, Christopher M Hovens^4^, Niall M Corcoran^4^, Clare Verrill^1,9^, Alastair D Lamb^1,2^

^1^Nuffield Department of Surgical Sciences, University of Oxford, UK

^2^Department of Urology, Oxford University Hospitals NHS Foundation Trust, Roosevelt Drive, Oxford, UK

^3^Bodleian Health Care Libraries, University of Oxford, UK

^4^Department of Surgery, Royal Melbourne Hospital, University of Melbourne, Melbourne, Australia

^5^Dana Farber Cancer Institute, Harvard, USA

^6^Division of Cancer Surgery^,^ Peter MacCallum Cancer Centre, Melbourne, Australia

^7^Sir Peter MacCallum Department of Oncology, University of Melbourne, Parkville, Australia.

^8^Weill Cornell Medical School, New York, USA

^9^NIHR Oxford Biomedical Research Centre, University of Oxford, John Radcliffe Hospital, Oxford, UK

**Contents:**

**Supplementary Methods (Page 2)**

Supplementary Methods 1. Search Strategy

**Supplementary Figures (Page 4)**

Supplementary Figure 1. Biochemical recurrence Forest plot

Supplementary Figure 2. Cancer-specific survival Forest plot

Supplementary Figure 3. Overall survival Forest plot

Supplementary Figure 4. Positive T1 stage Forest plot

Supplementary Figure 5. Positive T2 stage Forest plot

Supplementary Figure 6. Positive T3 stage Forest plot

Supplementary Figure 7. Positive T4 stage Forest plot

Supplementary Figure 8. Positive N status Forest plot

**Supplementary References (Page 6)**

**Supplementary Documents (Page 9)**

Supplementary Document 1. PROSPERO registration CRD42019122205

**Supplementary Tables (Attached as separate MS Excel Files)**

Supplementary Table 1. Summary of all papers included in systematic review.

Supplementary Table 2. Summary of Findings.

Supplementary Table 3. Risk of bias summary (ROBINS-E).

**Supplementary Material:**

**Supplementary Methods**

Supplementary Methods 1. Search strategy

Methodology

The following databases were searched from inception to 15 January 2019 (and were updated on 04/05/2020) for relevant studies: PubMed; Ovid EMBASE; the Cochrane Database of Systematic Reviews; SCOPUS; and the Web of Science (All Databases). The literature search combined the thesaurus terms Carcinoma, Ductal/ AND Prostatic Neoplasms/ where relevant, along with a set of phrases combined with OR to retrieve references, for example, about “ductal adenocarcinoma of the prostate”, “ductal carcinoma of the prostate”, or “ductal prostate cancer”. No limits were applied. The full search strategies are available in the appendix.

Search strategies designed and updated on 04/05/2020 by Eli Harriss, a librarian at the Bodleian Health Care Libraries, University of Oxford.

Search Results

|  | Search results 15/01/19 | Search results (2019-04/05/2020 only) |
| --- | --- | --- |
| PubMed | 344 | 20 |
| Ovid Embase | 234 | 24 |
| The Cochrane Database of Systematic Reviews | 0 | 0 |
| Web of Science – all databases | 260 | 28 |
| Scopus | 196 | 12 |
| Total | 1034 | 84 |
| Total after deduplication | 405 | 42 |
| Total after exclusion | 105 | 9 |

Search Strategies – 15/01/19

**Pubmed**

(ductal prostate cancer[Title/Abstract]) OR ((((((((((((((((("Prostatic Neoplasms"[Mesh]) AND "Carcinoma, Ductal"[Mesh])) OR ductal adenocarcinoma of the prostate[Title/Abstract]) OR ductal carcinoma of the prostate[Title/Abstract]) OR Prostatic ductal adenocarcinoma[Title/Abstract]) OR prostatic duct adenocarcinoma[Title/Abstract]) OR prostate duct adenocarcinoma[Title/Abstract]) OR prostate ductal adenocarcinoma[Title/Abstract]) OR papillary prostatic duct adenocarcinoma[Title/Abstract]) OR papillary adenocarcinoma of the prostate[Title/Abstract]) OR endometrioid adenocarcinoma of the prostate[Title/Abstract]) OR endometrial adenocarcinoma of the prostate[Title/Abstract]) OR endometrial carcinoma of the prostatic utricle[Title/Abstract]) OR endometrioid carcinoma of the prostatic utricle[Title/Abstract]) OR endometrial carcinoma of the uterus masculinus[Title/Abstract]) OR endometrioid carcinoma of the uterus masculinus[Title/Abstract])

**Embase**

1 "ductal adenocarcinoma of the prostate".mp. [mp=title, abstract, heading word, drug trade name, original title, device manufacturer, drug manufacturer, device trade name, keyword, floating subheading word, candidate term word]

2 "ductal carcinoma of the prostate".mp.

3 "Prostatic ductal adenocarcinoma".mp.

4 "ductal prostate cancer".mp.

5 "prostatic duct adenocarcinoma".mp.

6 "prostate duct adenocarcinoma".mp.

7 "prostate ductal adenocarcinoma".mp.

8 "papillary prostatic duct adenocarcinoma".mp.

9 "papillary adenocarcinoma of the prostate".mp.

10 "endometrioid adenocarcinoma of the prostate".mp.

11 "endometrial adenocarcinoma of the prostate".mp.

12 "endometrial carcinoma of the prostatic utricle".mp.

13 "endometrioid carcinoma of the prostatic utricle".mp.

14 "endometrial carcinoma of the uterus masculinus".mp.

15 "endometrioid carcinoma of the uterus masculinus".mp.

16 1 or 2 or 3 or 4 or 5 or 6 or 7 or 8 or 9 or 10 or 11 or 12 or 13 or 14 or 15 (227)

**Cochrane Database of Systematic Reviews**

Issue 1 of 12, January 2019

#1 MeSH descriptor: [Carcinoma, Ductal] explode all trees

#2 MeSH descriptor: [Prostatic Neoplasms] explode all trees

#3 #1 and #2

#4 "ductal adenocarcinoma of the prostate"

#5 "ductal carcinoma of the prostate"

#6 "Prostatic ductal adenocarcinoma"

#7 "ductal prostate cancer"

#8 "prostatic duct adenocarcinoma"

#9 "prostate duct adenocarcinoma"

#10 "prostate ductal adenocarcinoma"

#11 "papillary prostatic duct adenocarcinoma"

#12 "papillary adenocarcinoma of the prostate"

#13 "endometrioid adenocarcinoma of the prostate"

#14 "endometrial adenocarcinoma of the prostate"

#15 "endometrial carcinoma of the prostatic utricle"

#16 "endometrioid carcinoma of the prostatic utricle"

#17 "endometrial carcinoma of the uterus masculinus"

#18 "endometrioid carcinoma of the uterus masculinus"

#19 OR/#3-#18

**Web of Science**

1. TOPIC: ("ductal adenocarcinoma of the prostate") OR TITLE: ("ductal adenocarcinoma of the prostate")
2. TOPIC: ("ductal carcinoma of the prostate") OR TITLE: ("ductal carcinoma of the prostate")
3. TOPIC: ("Prostatic ductal adenocarcinoma") OR TITLE: ("Prostatic ductal adenocarcinoma")
4. TOPIC: ("ductal prostate cancer") OR TITLE: ("ductal prostate cancer")
5. TOPIC: ("prostatic duct adenocarcinoma") OR TITLE: ("prostatic duct adenocarcinoma")
6. TOPIC: ("prostate duct adenocarcinoma") OR TITLE: ("prostate duct adenocarcinoma")
7. TOPIC: ("prostate ductal adenocarcinoma") OR TITLE: ("prostate ductal adenocarcinoma")
8. TOPIC: ("papillary prostatic duct adenocarcinoma") OR TITLE: ("papillary prostatic duct adenocarcinoma")
9. TOPIC: ("papillary adenocarcinoma of the prostate") OR TITLE: ("papillary adenocarcinoma of the prostate")
10. TOPIC: ("endometrioid adenocarcinoma of the prostate") OR TITLE: ("endometrioid adenocarcinoma of the prostate")
11. TOPIC: ("endometrial adenocarcinoma of the prostate") OR TITLE: ("endometrial adenocarcinoma of the prostate")
12. TOPIC: ("endometrial carcinoma of the prostatic utricle") OR TITLE: ("endometrial carcinoma of the prostatic utricle")
13. TOPIC: ("endometrioid carcinoma of the prostatic utricle") OR TITLE: ("endometrioid carcinoma of the prostatic utricle")
14. TOPIC: ("endometrial carcinoma of the uterus masculinus") OR TITLE: ("endometrial carcinoma of the uterus masculinus")
15. TOPIC: ("endometrioid carcinoma of the uterus masculinus") OR TITLE: ("endometrioid carcinoma of the uterus masculinus")
16. #15 OR #14 OR #13 OR #12 OR #11 OR #10 OR #9 OR #8 OR #7 OR #6 OR #5 OR #4 OR #3 OR #2 OR #1

**Scopus**

( TITLE-ABS-KEY ( "endometrioid carcinoma of the uterus masculinus" ) )  OR  ( ( TITLE-ABS-KEY ( "ductal adenocarcinoma of the prostate" ) )  OR  ( TITLE-ABS-KEY ( "ductal carcinoma of the prostate" ) )  OR  ( TITLE-ABS-KEY ( "Prostatic ductal adenocarcinoma" ) )  OR  ( TITLE-ABS-KEY ( "ductal prostate cancer" ) ) )  OR  ( ( TITLE-ABS-KEY ( "prostatic duct adenocarcinoma" ) )  OR  ( TITLE-ABS-KEY ( "prostate duct adenocarcinoma" ) )  OR  ( TITLE-ABS-KEY ( "prostate ductal adenocarcinoma" ) ) )  OR  ( ( TITLE-ABS-KEY ( "papillary prostatic duct adenocarcinoma" ) )  OR  ( TITLE-ABS-KEY ( "papillary adenocarcinoma of the prostate" ) )  OR  ( TITLE-ABS-KEY ( "endometrioid adenocarcinoma of the prostate" ) ) )  OR  ( ( TITLE-ABS-KEY ( "endometrial adenocarcinoma of the prostate" ) )  OR  ( TITLE-ABS-KEY ( "endometrial carcinoma of the prostatic utricle" ) )  OR  ( TITLE-ABS-KEY ( "endometrioid carcinoma of the prostatic utricle" ) )  OR  ( TITLE-ABS-KEY ( "endometrial carcinoma of the uterus masculinus" ) ) )

**Supplementary Figures**

Supplementary Figure 1. Biochemical recurrence Forest plot


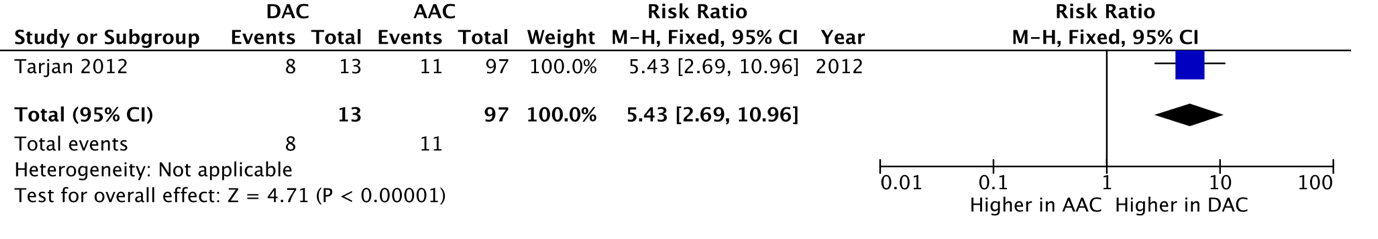


Supplementary Figure 2. Cancer-specific survival Forest plot


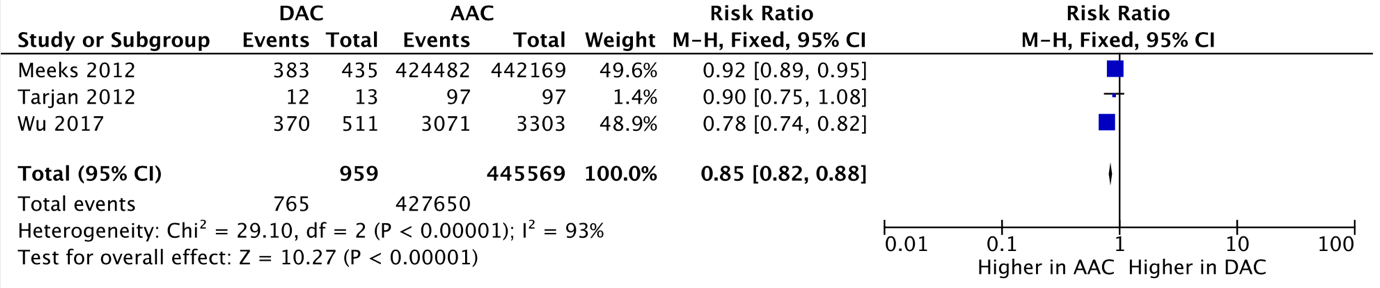


Supplementary Figure 3. Overall survival Forest plot


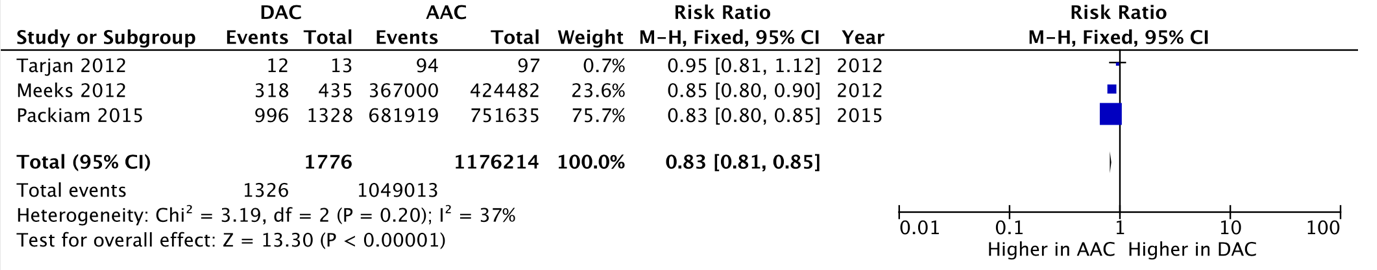


Supplementary Figure 4. Positive T1 stage Forest plot


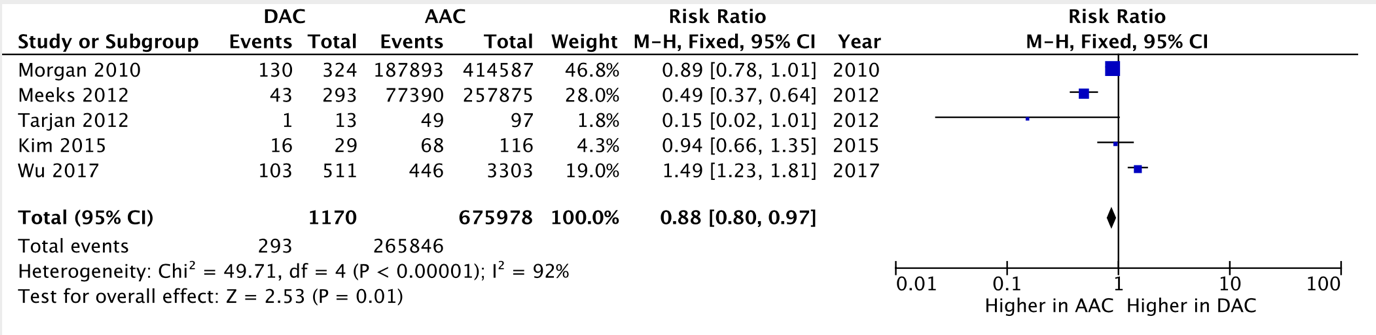


Supplementary Figure 5. Positive T2 stage Forest plot


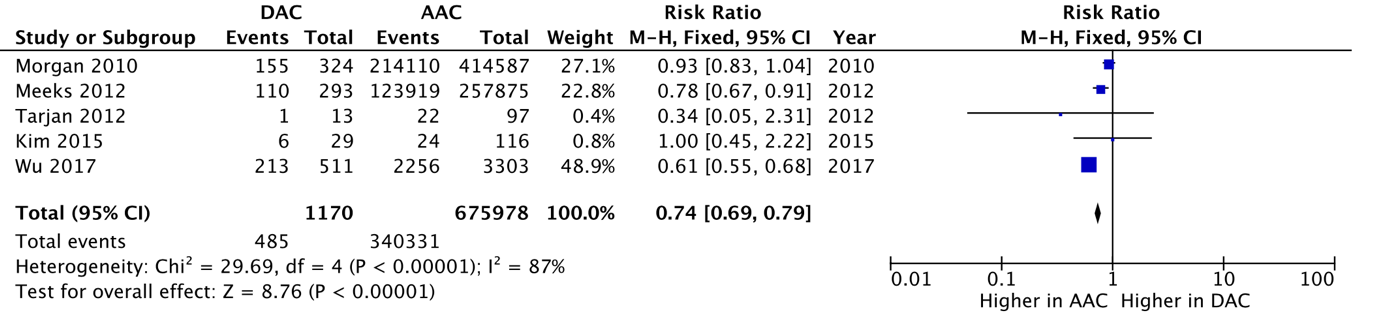


Supplementary Figure 6. Positive T3 stage Forest plot


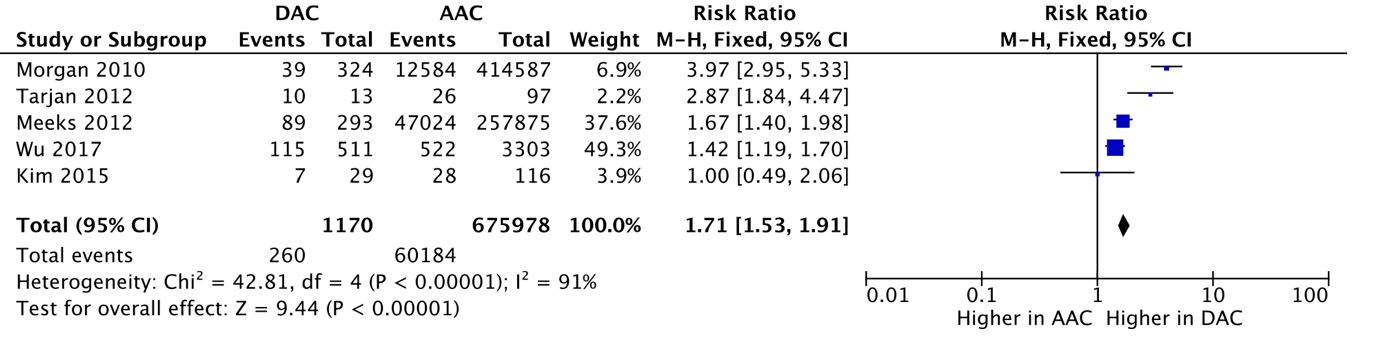


Supplementary Figure 7. Positive T4 stage Forest plot


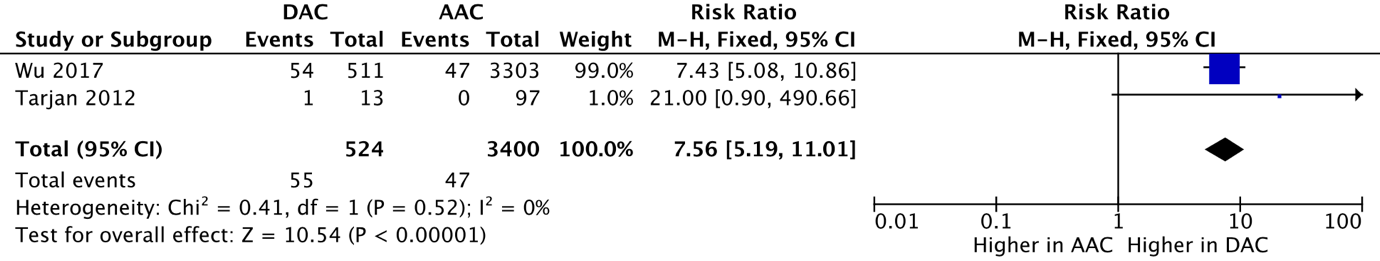


Supplementary Figure 8. Positive N status Forest plot


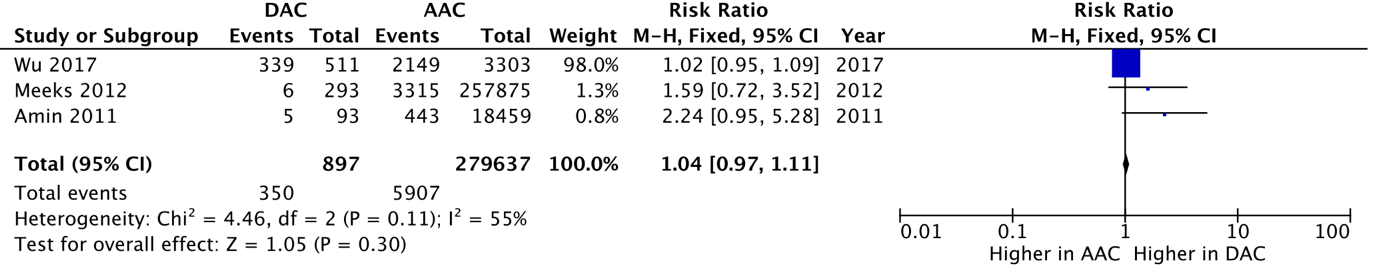


**Supplementary references**

67. Pratsinis, M. et al. Initial Diagnosis and Detection of Very Late Local Recurrence of a Ductal Prostate Cancer due to a Ureteral Stone. Case Rep. Urol. (2020).

68. Khanduri, S., Imam, T., Khan, M., Khan, S. & Khan, A. U. Rare Variant of Carcinoma Prostate Masquerading as Benign Prostatic Hyperplasia. Cureus 11, e4504 (2019).

69. Cakmak, S. et al. Prostatic ductal adenocarcinoma mimicking bladder cancer successfully treated with robotic radical prostatecectomy. Eur. Urol. Suppl. 18 (6), e2719 (2019).

70. Merrett, C., Gross, T., Moran, D. & Zargar, H. Local recurrence of prostatic ductal adenocarcinoma despite clear surgical margins. Urol. case reports 17, 65–66 (2018).

71. Khan, M. et al. Isolated prostate cancer soft tissue recurrence 10 years after radical prostatectomy: A case report. BJU Int. 119, 106 (2017).

72. Yadav, S. S., Baghel, P., Tomar, V., Agarwal, N. & Dhakad, D. Prostatic Ductal Adenocarcinoma—A Rare Entity With Radiological Dilemma Demanding Endoscopic Biopsy. Urology 108, e1–e2 (2017).

73. Wang, Y. G., Davies, N., Desai, D. & Yap, H.-W. Local control of isolated anterior urethral metastasis from ductal prostate cancer. J. Clin. Urol. 10, 309–310 (2017).

74. Washburn, E. R., Weyant, G. W., Yang, X. J. & Yang, Z. A rare case of prostatic ductal adenocarcinoma presenting as papillary metastatic carcinoma of unknown primary: A case report and review of the literature. Hum. Pathol. Case Reports 6, 26–31 (2016).

75. Ramasamy, K., Khor, T. W., Tham, T. M. & Tan, C. K. Prostate ductal adenocarcinoma. BJU Int. 118, 36 (2016).

76. Tavukcu, H. H. et al. Ductal Adenocarcinoma of the Prostate With a Rare Clinical Presentation; Late Gastric Metastasis. Urol. case reports 7, 28–30 (2016).

77. Ji, C. et al. Advanced prostatic ductal carcinoma in a patient with a long survival time following a total pelvis exenteration: A case report. Oncol. Lett. 11, 1509–1511 (2016).

78. Hayashi, Y. et al. Ductal Adenocarcinoma of the Prostate: A Case Report. Case Rep. Oncol. 9, 802–805 (2016).

79. Kamiyama, Y. et al. Chemotherapy with Gemcitabine and Cisplatin for Advanced Ductal Adenocarcinoma of the Prostate: Clinical Courses of Two Patients. Tohoku J. Exp. Med. 237, 317–321 (2015).

80. Fujiwara, R. et al. Metastatic Prostatic Ductal Adenocarcinoma Successfully Treated with Docetaxel Chemotherapy: A Case Report. Case Rep. Oncol. 8, 339–344 (2015).

81. Hardie, A. D., Naveed, M. A. & Clarke, H. S. Ductal prostatic adenocarcinoma: magnetic resonance imaging documenting the effect of hormone-radiotherapy. J. Coll. Physicians Surg. Pak. 24 Suppl 1, S55-6 (2014).

82. Ogawa, S. et al. The probability of involvement of human papillomavirus in the carcinogenesis of bladder small cell carcinoma, prostatic ductal adenocarcinoma, and penile squamous cell carcinoma: a case report. BMC Res. Notes 7, 909 (2014).

83. Niwa, N. et al. Ductal adenocarcinoma of the prostate forming a mass in the retrovesical space. Urology 84, e9-10 (2014).

84. Linden-Castro, E. et al. Abiraterone acetate and castration resistant ductal adenocarcinoma of the prostate. Case Rep. Urol. 2014, 508305 (2014).

85. Torricelli, F. C. M., Tucherman, M., Melogno, R. & Coelho, R. F. Large cystic ductal carcinoma of the prostate: imaging findings and minimally invasive surgical treatment. BMJ Case Rep. 2014, (2014).

86. Escarpa, S. et al. A case report: Ductal adenocarcinoma of the prostate. Reports Pract. Oncol. Radiother. 18, S309–S310 (2013).

87. Stajno, P., Kalinowski, T., Ligaj, M. & Demkow, T. An incidentally diagnosed prostatic ductal adenocarcinoma. Cent. Eur. J. Urol. 66, 164–167 (2013).

88. Shaaban, H., Thomas, D. & Guron, G. A rare case of metastatic ductal type prostate adenocarcinoma presenting with syndrome of inappropriate secretion of antidiuretic hormone: a case report and review. J. Cancer Res. Ther. 8, 308–310 (2012).

89. Anila, K. R., Somanathan, T., Mathews, A. & Jayasree, K. An unusual variant of prostatic adenocarcinoma with metastasis to testis. A case report. Gulf J. Oncolog. 73–76 (2012).

90. Henderson-Jackson, E. et al. Cystic prostatic ductal adenocarcinoma: an unusual presentation and cytological diagnosis. Ann. Clin. Lab. Sci. 42, 81–88 (2012).

91. Sfoungaristos, S. et al. An 82-year-old Caucasian man with a ductal prostate adenocarcinoma with unusual cystoscopic appearance: a case report. J. Med. Case Rep. 5, 4 (2011).

92. Loghin, A., Dema, A., Taban, S., Preda, O. & Borda, A. Early micropapillary prostatic ductal adenocarcinoma mimicking nephrogenic adenoma. Virchows Arch. 459, S285–S285 (2011).

93. Kumar, A. & Mukherjee, S. D. Metastatic ductal carcinoma of the prostate: a rare variant responding to a common treatment. Can. Urol. Assoc. J. = J. l’Association des Urol. du Canada 4, E50-4 (2010).

94. Paterson, C., Correa, P. D. & Russell, J. M. Ductal Variant of Adenocarcinoma Prostate Responding to Docetaxel - a Case Report. Clin. Oncol. 22, 617 (2010).

95. Jung, H. et al. Prostatic ductal adenocarcinoma. Korean J. Urol. 50, 404–407 (2009).

96. Tu, W. H., Jensen, K., Freiha, F. & Liao, J. C. A case of prostatic adenocarcinoma recurrence presenting as ductal carcinoma of the prostate. Nat. Clin. Pract. Urol. 5, 55–58 (2008).

97. Lim, T. et al. Thyroid transcription factor-1 may be expressed in ductal adenocarcinoma of the prostate:a potential pitfall. J. Clin. Pathol. 60, 941–943 (2007).

98. Izumi, K. et al. A case of ductal carcinoma of the prostate after transurethral resection of prostate. Acta Urol. Jpn. 53, 315–318 (2007).

99. Kato, T. et al. Carbohydrate antigen 19-9-positive prostatic ductal adenocarcinoma effectively treated with cisplatin and gemcitabine. Int. J. Urol. 14, 1103–1106 (2007).

100. Sato, K., Tachibana, H., Tsuzuki, T., Ueda, Y. & Katsuda, S. Prostatic ductal adenocarcinoma mimicking villous adenoma of the urethra. Virchows Archiv : an international journal of pathology 449, 597–599 (2006).

101. Green, J. M., Tang, W. W., Jensen, B. W. & Orihuela, E. Isolated recurrence of ductal prostate cancer to anterior urethra. Urology 68, 428.e13–5 (2006).

102. Suzuki, T. et al. Prostatic duct adenocarcinoma with pagetoid spread on the glans penis: A case report. Acta Urol. Jpn. 52, 887–890 (2006).

103. Alrahwan, D., Staerkel, G. & Gong, Y. Fine needle aspiration cytology of a metastatic duct carcinoma of the prostate: a case report. Acta Cytol. 50, 469–472 (2006).

104. Yamashita, S., Inaba, Y., Soma, F. & Katayama, Y. Pure prostatic papillary adenocarcinoma with ductal features. Hinyokika Kiyo. 51, 207–9; discussion 210 (2005).

105. Tulunay, O., Orhan, D., Baltaci, S., Gogus, C. & Muftuoglu, Y. Z. Prostatic ductal adenocarcinoma showing Bcl-2 expression. Int. J. Urol. 11, 805–808 (2004).

106. Iguchi, T. et al. Effective combined chemotherapy for prostatic ductal adenocarcinoma: continuous venous infusion of 5-fluorouracil and low-dose consecutive cisplatin. BJU Int. 92 Suppl 3, e54–e55 (2003).

107. Matsui, Y. et al. Ductal adenocarcinoma of the prostate associated with prostatic multilocular cyst. Int. J. Urol. 9, 413–415 (2002).

108. Rubinowicz, D. M., Soloway, M. S., Lief, M. & Civantos, F. Hemospermia and expressed tumor in the urethra: An unusual presentation of ductal carcinoma of the prostate. J. Urol. 163, 915 (2000).

109. Vandersteen, D. P., Wiemerslage, S. J. & Cohen, M. B. Prostatic duct adenocarcinoma: a cytologic and histologic case report with review of the literature. Diagn. Cytopathol. 17, 480–483 (1997).

110. Elgamal, A. A. et al. Exophytic papillary prostatic duct adenocarcinoma with endometrioid features, occurring in prostatic urethra after TURP. Urology 43, 737–742 (1994).

111. Daugherty, R. L., Cos, L. R., Keller, J. W. & di Sant’Agnese, A. Nondetectable prostate-specific antigen in moderately differentiated adenocarcinoma of prostate. Urology 39, 552–555 (1992).

112. Kullu, S., Ersev, A., Simsek, F. & Ersev, D. Adenocarcinoma of the prostate with endometrioid features. Int. Urol. Nephrol. 23, 577–580 (1991).

113. Odom, D. G., Westphal, K. W., Hawksley, V. C. & Deshon, G. E. Endometrioid carcinoma of prostate. Urology 31, 217–219 (1988).

114. Das, S. Endometrial carcinoma of prostate. Urology 27, 543–545 (1986).

115. Cia, E. M. M. et al. Metastases in epididymides from papillary adenocarcinoma of prostate. Urology 18, 607–608 (1981).

116. Kauder, D. H. Endometrial carcinoma of prostatic utricle. Urology 10, 272–275 (1977).

117. Novicki, D. E. & Gehring, G. G. Urethral carcinoma after radiation therapy for mixed prostatic carcinoma. J. Urol. 116, 122–123 (1976).

118. Scott, M. B., Goldstein, A. M., Onofrio, R. C. & Cosgrove, M. D. Papillary adenocarcinoma of prostate. Urology 8, 227–230 (1976).

119. Merchant Jr., R. F., Graham, A. R., Bucher, W. C. & Parker, D. A. Endometrial carcinoma of prostatic utricle with osseous metastases. Urology 8, 169–173 (1976).

120. Young, B. W. Endometrial (papillary) carcinoma of the prostatic utricle - response to orchiectomy. A case report. Cancer 32, 1293–1300 (1973).

121. Chandra, A., Dasgupta, P., Elhage, O., Frydenberg, M. & Harkin, T. Clinical outcomes and morphometry of prostate ductal adenocarcinoma: the importance of ductal proportions. BJU Int. 121, 49–50 (2018).

122. Fedorina, T. & Poletaeva, S. Diagnosis of prostate ductal adenocarcinoma in needle biopsy. Virchows Arch. 465, S157–S157 (2014).

123. Kan, R. W. M. et al. Ductal adenocarcinoma of the prostate: a Hong Kong case series. Int. Urol. Nephrol. 46, 2133–2137 (2014).

124. Sha, J. et al. Ductal adenocarcinoma of the prostate: Immunohistochemical findings and clinical significance. Onco. Targets. Ther. 6, 1501–1506 (2013).

125. Menendez, C. L. et al. Mixed peripheral ductal prostatic adenocarcinoma in radical prostatectomies: A five-year experience in a small Spanish Community Hospital. Virchows Arch. 457, 241 (2010).

126. Watanabe, J. et al. [Papillary adenocarcinoma of the prostate: report of 3 cases]. Hinyokika Kiyo. 46, 273–276 (2000).

127. Oxley, J. D., Abbott, C. D., Gillatt, D. A. & MacIver, A. G. Ductal carcinomas of the prostate: a clinicopathological and immunohistochemical study. Br. J. Urol. 81, 109–115 (1998).

128. Millar, E. K., Sharma, N. K. & Lessells, A. M. Ductal (endometrioid) adenocarcinoma of the prostate: a clinicopathological study of 16 cases. Histopathology 29, 11–19 (1996).

129. Lemberger, R. J., Bishop, M. C., Bates, C. P., Blundell, W. & Ansell, I. D. Carcinoma of the prostate of ductal origin. Br. J. Urol. 56, 706–709 (1984).

**Supplementary Documents**

Supplementary Document 1. PROSPERO registration CRD42019122205

| **PROSPERO** |
| --- |
| **International prospective register of systematic reviews**  Ductal Adenocarcinoma of the Prostate (DAC): a comprehensive systematic review and meta-analysis of incidence, presentation and management *Nithesh Ranasinha, Altan Omer, Eli Harris, Clare Verrill, Ken Chow, Paolo Chetta, Andrew Erickson, Ian Mills, Richard Bryant, Freddie Hamdy, Declan Murphy, Massimo Loda, Chris Hovens, Niall Corcoran, Alastair Lamb*  Citation  Nithesh Ranasinha, Altan Omer, Eli Harris, Clare Verrill, Ken Chow, Paolo Chetta, Andrew Erickson, Ian Mills, Richard Bryant, Freddie Hamdy, Declan Murphy, Massimo Loda, Chris Hovens, Niall Corcoran, Alastair Lamb. Ductal Adenocarcinoma of the Prostate (DAC): a comprehensive systematic review and meta-analysis of incidence, presentation and management. PROSPERO 2019 CRD42019122205 Available from: https://www.crd.york.ac.uk/prospero/display_record.php?ID=CRD42019122205  Review question  How can we define ductal adenocarcinoma of the prostate?  How can we diagnose ductal adenocarcinoma of the prostate?  What is the optimal management strategy for ductal adenocarcinoma of the prostate?  How does ductal adenocarcinoma of the prostate progress?  What is the survival of ductal adenocarcinoma of the prostate?  Searches  The following databases were searched from inception to 15 January 2019 for relevant studies: PubMed; Ovid EMBASE; the Cochrane Library; Scopus; and the Web of Science Core Collection. The literature search combined the thesaurus terms Carcinoma, Ductal/ AND Prostatic Neoplasms/ where relevant, along with a set of phrases combined with OR to retrieve references, for example, about “ductal adenocarcinoma of the prostate”, “ductal carcinoma of the prostate”, or “ductal prostate cancer”. We did not use any limits.  Types of study to be included  There are no restrictions on the types of study design eligible for inclusion, provided primary data was provided. the majority of studies are retrospective, descriptive case series or case reports.  Condition or domain being studied  Ductal adenocarcinoma (DAC) is relatively rare, but is nonetheless the second most common subtype of prostate cancer. First described in 1967, opinion is still divided regarding its biology, prognosis and outcome. We systematically interrogated the literature in order to clarify the epidemiology, diagnosis, management, progression and survival of DAC.  Participants/population  The population of interest is men with suspected prostate cancer. Inclusion criteria include primary case series or reports of ductal prostate cancer.  The excluded studies met at least one of the following criteria: (i) the article was a review or meta-analysis, editorial comment, letter or book chapter (ii) non-English language (iii) a basic science cancer biology article (iv) couldn’t be found online.  Intervention(s), exposure(s)  We systematically interrogated the literature in order to clarify the epidemiology, diagnosis, management, progression and survival of prostate ductal adenocarcinoma.  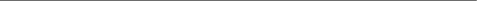  *Page: 1 / 4* |


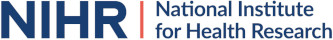


| **PROSPERO** |
| --- |
| **International prospective register of systematic reviews**  Comparator(s)/control  Where relevant, acinar adenocarcinoma, the main subtype of prostate cancer, was used as a comparator.  Context  Studies were included if they met the following criteria: (i) reports of a case/s of prostate ductal adenocarcinoma.  The excluded studies met at least one of the following criteria: (i) the article was a review or meta-analysis, editorial comment, letter or book chapter (ii) non-English language (iii) a basic science cancer biology article (iv) could not be found online.  Main outcome(s)  Incidence: DAC incidence  Diagnosis: proportion of DAC diagnosed pre radical treatment and post radical treatment, PSA at presentation, TNM stage at presentation, proportion of metastatic cases at presentation.  Treatment: Optimal treatment modalities for localised and metastatic DAC Outcome: Cancer specific survival, overall survival and time to biochemical relapse  Timing and effect measures  Comparative analysis of outcomes is limited to studies with 5-year follow-up periods.  Additional outcome(s)  None.  Timing and effect measures  Not applicable.  Data extraction (selection and coding)  The data were extracted from full-length articles (and abstracts where full-length articles were unavailable) by two reviewers, NR and AO. Where discrepancies arose, a supervisor, AL, was consulted. The data included the author’s name, city and country, the year and journal of publication, number of DAC cases reported (with comparative AAC cases where available) and number of pure versus mixed cases, time period covered, a pathological definition of the tumour, the patients’ age, Gleason score and PSA, method of DAC diagnosis, metastases on initial presentation, primary treatment modality and post-treatment outcomes (including post-treatment metastases and results of follow-up treatments).  Risk of bias (quality) assessment  Two independent assessors will review the title, abstract and full articles of the retrieved papers to assess their eligibility according to defined inclusion and exclusion criteria. Any discrepancies in assessors’ selections will be resolved in a meeting between assessors and lead supervisor. Corresponding authors of selected studies will be contacted for further information where necessary.  The Cochrane Risk of Bias ROBINS tool will be used to assess eligible articles for risk of bias in 4 domains: (i) patient selection, (ii) index test, (iii) reference standard and (iv) flow and timing.  Strategy for data synthesis  For the case series an aggregate approach to synthesis will be used. For the case reports a descriptive synthesis will be used. A quantitive synthesis will be used if the included studies are sufficiently homogenous. Risk ratio will be used to analyse dichotomous outcomes. Statistical heterogeneity will be evaluated using the ?2 test with a p value of <0.10 indicating heterogeneity.  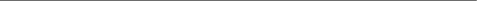  *Page: 2 / 4* |


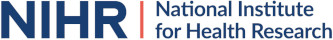


| **PROSPERO** |
| --- |
| **International prospective register of systematic reviews**  Analysis of subgroups or subsets  Subgroup analysis will separate treatment of localised disease from metastatic disease. Subgroup analysis of pure DAC and mixed DAC will also be performed.  Contact details for further information  Nithesh Ranasinha nithesh.ranasinha@jesus.ox.ac.uk  Organisational affiliation of the review  Nuffield Department of Surgical Sciences, University of Oxford  Review team members and their organisational affiliations  Mr Nithesh Ranasinha. University of Oxford Mr Altan Omer. Nuffield Department of Surgical Sciences, University of Oxford Ms Eli Harris. Nuffield Department of Surgical Sciences, University of Oxford Assistant/Associate Professor Clare Verrill. Nuffield Department of Surgical Sciences, University of Oxford Ken Chow. Epworth Prostate Centre, Melbourne, Australia Paolo Chetta. Dana Farber Cancer Institute, Harvard, USA Andrew Erickson. Nuffield Department of Surgical Sciences, University of Oxford Ian Mills. Nuffield Department of Surgical Sciences, University of Oxford Professor Richard Bryant. Nuffield Department of Surgical Sciences, University of Oxford Professor Freddie Hamdy. Nuffield Department of Surgical Sciences, University of Oxford Declan Murphy. Peter MacCallum Cancer Centre, Melbourne, Australia Massimo Loda. Dana Farber Cancer Institute, Harvard, USA Chris Hovens. Epworth Prostate Centre, Melbourne, Australia Niall Corcoran. Epworth Prostate Centre, Melbourne, Australia Mr Alastair Lamb. Nuffield Department of Surgical Sciences, University of Oxford  Type and method of review  Diagnostic, Epidemiologic, Intervention, Meta-analysis, Prognostic, Systematic review  Anticipated or actual start date  02 May 2018  Anticipated completion date  30 September 2019  Funding sources/sponsors  None  Conflicts of interest Language  English  Country  England  Stage of review  Review Ongoing  Subject index terms status  Subject indexing assigned by CRD  Subject index terms  Carcinoma, Ductal; Humans; Incidence; Male; Prostatic Neoplasms  Date of registration in PROSPERO  16 August 2019  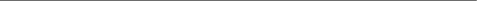  *Page: 3 / 4* |


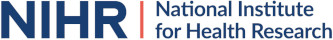


| **PROSPERO** |
| --- |
| Date of publication of this version  16 August 2019  Details of any existing review of the same topic by the same authors Stage of review at time of this submission  **Stage**  Preliminary searches Piloting of the study selection process Formal screening of search results against eligibility criteria Data extraction Risk of bias (quality) assessment Data analysis  Versions  **Started Completed**  Yes No Yes Yes Yes Yes No No Yes No No No  **International prospective register of systematic reviews**  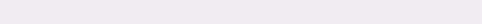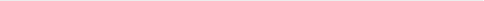  PROSPERO This information has been provided by the named contact for this review. CRD has accepted this information in good faith and registered the review in PROSPERO. The registrant confirms that the information supplied for this submission is accurate and complete. CRD bears no responsibility or liability for the content of this registration record, any associated files or external websites.  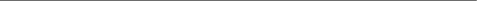  Powered by TCPDF (www.tcpdf.org)  16 August 2019  *Page: 4 / 4* |


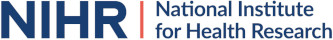


**Supplementary Tables**

Supplementary Table 1. Summary of all papers included in systematic review.

MS Excel file submitted as separate supplementary file titled ‘Supplementary Table 1’

Supplementary Table 2. Summary of Findings.

MS Excel file submitted as separate supplementary file titled ‘Supplementary Table 2’

Supplementary Table 3. Risk of bias summary (ROBINS-E).

MS Excel file submitted as separate supplementary file titled ‘Supplementary Table 3’
